# Nano-silica particles synergistically IgE-mediated mast cell activation exacerbating allergic inflammation in mice

**DOI:** 10.3389/fimmu.2022.911300

**Published:** 2022-07-22

**Authors:** Yong-Shi Yang, Meng-Da Cao, An Wang, Qing-Mei Liu, Dan-Xuan Zhu, Ying Zou, Ling-Ling Ma, Min Luo, Yang Shao, Dian-Dou Xu, Ji-Fu Wei, Jin-Lyu Sun

**Affiliations:** ^1^ Department of Allergy, State Key Laboratory of Complex Severe and Rare Diseases, Peking Union Medical College Hospital, Chinese Academy of Medical Science and Peking Union Medical College, Beijing, China; ^2^ Research Division of Clinical Pharmacology, the First Affiliated Hospital of Nanjing Medical University, Nanjing, China; ^3^ Beijing Engineering Research Center of Radiographic Techniques and Equipment, Institute of High Energy Physics, Chinese Academy of Sciences, Beijing, China; ^4^ Women and Children Central Laboratory, The First Affiliated Hospital of Nanjing Medical University, Nanjing, China; ^5^ Shanghai Institute of Applied Physics, Chinese Academy of Sciences, Shanghai, China; ^6^ Shanghai Advanced Research Institute, Chinese Academy of Sciences, Shanghai, China; ^7^ Department of Pharmacy, Jiangsu Cancer Hospital, Jiangsu Institute of Cancer Research, The Affiliated Cancer Hospital of Nanjing Medical University, Nanjing, China

**Keywords:** allergic asthma, smog particles, mast cell, MAPK, silica nanoparticles

## Abstract

**Background:**

Allergic respiratory diseases have increased dramatically due to air pollution over the past few decades. However, studies are limited on the effects of inorganic components and particulate matter with different particle sizes in smog on allergic diseases, and the possible molecular mechanism of inducing allergies has not been thoroughly studied.

**Methods:**

Four common mineral elements with different particle sizes in smog particles were selected, including Al_2_O_3_, TiO_2_, Fe_2_O_3_, and SiO_2_. We studied the relationship and molecular mechanism of smog particle composition, particle size, and allergic reactions using mast cells, immunoglobulin E (IgE)-mediated passive cutaneous anaphylaxis (PCA) model, and an ovalbumin (OVA)-induced asthmatic mouse model *in vitro* and *in vivo*, combined with transmission electron microscopy, scanning transmission X-ray microscopy analysis, and transcriptome sequencing.

**Results:**

Only 20 nm SiO_2_ particles significantly increased β-hexosaminidase release, based on dinitrophenol (DNP)-human serum albumin (HSA) stimulation, from IgE-sensitized mast cells, while other particles did not. Meanwhile, the PCA model showed that Evan’s blue extravasation in mice was increased after treatment with nano-SiO_2_ particles. Nano-SiO_2_ particles exposure in the asthmatic mouse model caused an enhancement of allergic airway inflammation as manifested by OVA-specific serum IgE, airway hyperresponsiveness, lung inflammation injury, mucous cell metaplasia, cytokine expression, mast cell activation, and histamine secretion, which were significantly increased. Nano-SiO_2_ particles exposure did not affect the expression of FcϵRI or the ability of mast cells to bind IgE but synergistically activated mast cells by enhancing the mitogen-activated protein kinase (MAPK) signaling pathway, especially the phosphorylation levels of the extracellular signal-regulated kinase (ERK)1/2. The ERK inhibitors showed a significant inhibitory effect in reducing β-hexosaminidase release.

**Conclusion:**

Our results indicated that nano-SiO_2_ particles stimulation might synergistically activate IgE-sensitized mast cells by enhancing the MAPK signaling pathway and that nano-SiO_2_ particles exposure could exacerbate allergic inflammation. Our experimental results provide useful information for preventing and treating allergic diseases.

## Introduction

Due to environmental changes, air pollutants, and aeroallergens, the morbidity, and prevalence of allergic respiratory diseases have increased dramatically over the past few decades (1, 2). In addition to lifestyle changes, many epidemiological and experimental studies explicitly highlight the role of air pollution in the development of allergic disease and increase the frequency of emergency department visits and hospitalizations for asthma (3–5). Importantly, pollutants can act as adjuvants to potentiate the development of pollen allergies, and early-life exposure may increase the risk of aeroallergen sensitization (6, 7).

Undoubtedly, air pollution has become a severe threat to global health. According to the World Health Organization (WHO) (https://www.who.int/), ambient air pollution accounts for an estimated 4.2 million deaths per year, and over 90% of people live in places where air quality levels do not reach WHO guideline levels. Air pollution has been of great concern over the last decade in China due to the increasing hazardous dense smog affecting most parts of the country (8). Among the various air pollutants, which are composed of gases (O_3_, NO_2_, CO, and SO_2_) and particulate matter (PM), PM causes the most significant harmful effects during a smog episode (9). Fine particulate matter (PM2.5), one of the main components of smog particles, refers to particulate matter with an aerodynamic diameter ≤ 2.5 μm, which is an extraordinarily complex mixture of diverse chemical, physical and biological components with a wide range of morphological, chemical, physical, and thermodynamic characteristics (10). The composition of smog particles includes not only a large number of biologically active substances, such as pollen, fungal spores, and microorganisms, but also nonbiologically active substances, such as heavy metals, transition metals, organic acids, and polycyclic aromatic hydrocarbons, which are the carriers and catalysts of many pollutants (11, 12). However, which components of smog particles play a significant role in developing allergic diseases have not yet been fully studied.

Mast cells are one of the critical effector cells in allergic diseases and are predominantly localized at sites in the skin, mucous membranes, airways, and intestine where they contact the environment; therefore, mast cells are considered to be the sentinel cells for environmental insults (13). Mast cells can recognize immunological, inflammatory, and environmental factors by various types of receptors, including high-affinity immunoglobulin E (IgE) receptors (FcϵRI), G protein-coupled receptors, or ion channels, and release bioactive mediators through IgE pathways or non-IgE pathway activation (14). Previous studies on the effects of smog particles on allergic diseases have primarily focused on complex mixtures. For example, Piao et al. reported that PM2.5 exposure could exacerbate oxidative stress and enhance PM2.5 phagocytosis by activating the NF-κB signaling pathway in an ovalbumin (OVA)-induced allergic rhinitis mouse model (15). Another study found that environmentally relevant metal and transition metal ions, such as Al^3+^, Cd^2+^, Sr^2+^, and Ni^2+^, could increase the level of allergen-mediated mast cell activation, which indicated that nonbiologically active substances in smog particles also played an essential role in allergic diseases (12).

However, studies on the effects of inorganic components and particulate matter with different particle sizes in smog particles on allergic diseases are limited, and the possible molecular mechanism of inducing allergies is not completely clear. In our previous study, we chose nine typical water-soluble inorganic salts and three water-soluble organic acids in PM2.5 from Beijing to investigate the cytotoxicity and activation effect on mast cells. The results showed that only malonic acid had a very slight activation effect on mast cells *via* the IgE pathway (16). The source and composition of smog particles are very complex. Elemental analysis shows that mineral elements and metal elements, such as Na, Fe, Al, Si, Ti, K, Mg, Ca, and Mn, are the main constituent elements (17, 18). In the present study, limited by the source of the material, we only selected four common mineral elements with different particle sizes in smog particles, including aluminum oxide (Al_2_O_3_, 20 nm and 26.93 μm), titanium oxide (TiO_2_, 20 nm and 1 μm), iron oxide (Fe_2_O_3_, 50 nm and 1 μm) and silicon dioxide (SiO_2_, 20 nm and 2.34 μm). We studied the relationship and molecular mechanism of smog particle composition, particle size, and allergic effect using mast cells, IgE-mediated passive cutaneous anaphylaxis (PCA) model, and an OVA-induced asthmatic mouse model *in vitro* and *in vivo*. Finally, we found that nano-SiO_2_ particles stimulation might synergistically activate IgE-sensitized mast cells by enhancing the mitogen-activated protein kinase (MAPK) signaling pathway and that nano-SiO_2_ particles exposure could exacerbate allergic inflammation.

## Materials and methods

### Reagents and antibodies

Aluminum oxide (Al_2_O_3_, 20 nm and 26.93 μm), titanium oxide (TiO_2_, 20 nm and 1 μm), iron oxide (Fe_2_O_3_, 50 nm and 1 μm) and silicon dioxide (SiO_2_, 20 nm and 2.34 μm) were purchased from Shanghai YunFu Nanotechnology Co., Ltd. (Shanghai, China), Fuchen Chemical Reagent Co., Ltd. (Tianjin, China), and Aladdin (Shanghai, China). Monoclonal anti-dinitrophenol (DNP)-IgE antibody, DNP-human serum albumin (HSA), and 4-nitrophenyl N-acetyl-β-D-glucosaminide were obtained from Sigma–Aldrich (St. Louis, USA). Antibodies as against phosphor-JNK, JNK, phosphor-LYN, LYN, phosphor-Plcγ1, Plcγ1, phosphor-P38, P38, phosphor-ERK1/2, ERK1/2, NF-κB, and β-actin were obtained from Cell Signaling Technology Inc. (Danvers, MA). ERK inhibitor U0126-EtOH and p38 inhibitor SB203580 were purchased from MedChem Express (Monmouth Junction, NJ, USA), PE-anti-mouse CD117 (c-kit), APC-anti-mouse FcϵRIα, and FITC-anti-mouse IgE antibodies were purchased from Biolegend (San Diego, CA, USA). Recombinant interleukin (IL)-3 and stem cell factor (SCF) were purchased from PeproTech (Cranbury, NJ, USA). Evans blue and formamide were obtained from Dalian Meilun Biotechnology Co. Ltd. (Dalian, China). The ovalbumin (OVA; grade V) was purchased from Sigma Company (St. Louis, USA), and aluminum hydroxide was purchased from Thermo Fisher Scientific (Massachusetts, USA).

### BMMC culture

Mouse bone marrow-derived mast cells (BMMCs) were isolated from BALB/c mouse femurs and cultured in complete RPMI-1640 with 100 U/mL penicillin, 100 U/mL streptomycin, 2 mM L-glutamine, 0.1 mM nonessential amino acids, 10% foetal bovine serum, 10 ng/mL IL-3, and 10 ng/mL SCF. After 4-6 weeks in culture, mast cell purity was evaluated by flow cytometry detection of cell-surface CD117 and FcϵRI expression.

### Cell viability assay

The BMMCs were seeded in 96-well plates at a density of 1×10^5^ cells/well and then were subsequently treated with Al_2_O_3_ (20 nm and 26.93 μm), TiO_2_ (20 nm and 1 μm), Fe_2_O_3_ (50 nm and 1 μm), and SiO_2_ (20 nm and 2.34 μm) at concentrations of 0, 50, 100, and 200 μg/mL for 1 hour. Cell viability was measured with the Cell Counting Kit 8 (CCK8) (Dojindo Laboratories, Tokyo, Japan) according to the manufacturer’s protocol.

### Measurement of mast cell degranulation in the IgE and non-IgE Pathways

For the IgE pathway, the BMMCs were sensitized with 500 ng/mL mouse anti-DNP IgE for 12 hours and then washed with Tyrode’s buffer before being dispensed into 96-well plates. For treated samples, the particles were applied for 1 hour. The molecule inhibitors (ERK inhibitor U0126-EtOH 5 μM/L, and p38 inhibitor SB203580 10 μM/L), were also applied for 1 hour. Subsequently, after stimulation with 100 ng/mL DNP-HSA for 30 min, the supernatant and cell lysate were reacted for 1.5 hours with 1 mM 4-nitrophenyl-N-acetyl-β-D-glucosaminide at 37 °C. The reaction was stopped with 0.2 M glycine solution. The optical density (OD) at 405 nm was measured with Multiskan GO (Thermo Fisher Scientific, Massachusetts, USA). For the non-IgE pathway, the BMMCs were not sensitized with mouse anti-DNP IgE and directly treated with the particles for 1 hour after seeding in 96-well plates. Compound 48/80 (Sigma–Aldrich, St. Louis, USA) served as the positive control. The β-hexosaminidase (β-hex) release was evaluated using the following formula: β-hex release (%) = absorbance of supernatant/(absorbance of supernatant + absorbance of cell lysates) × 100%.

### Experimental procedure for animal experiments

Female BALB/c mice, 6-8 weeks old, were purchased from Charles River Laboratories (Beijing, China) and used after 1 week of quarantine and acclimatization. All mice were maintained at conventional animal facilities under standard conditions. All animal use procedures were conducted in accordance with the National Institutes of Health and approved by Nanjing Medical University’s Institutional Animal Care and Use Committee (IACUC-2007037).

For the IgE-mediated PCA model, 20 female BALB/c mice were randomly divided into four groups: blank group, SiO_2_ group, DNP-HSA group, and DNP-HSA+SiO_2_ group. Briefly, BALB/c mice were injected intradermally with 500 ng of anti-DNP IgE into the left ear. After a 24-hour infiltration period, 200 μl of 5 mg/ml Evan’s blue solution containing 100 μg DNP-HSA and/or 10 mg/kg nano-SiO_2_ particles (about 200 μg/per mouse) was administered into the tail vein. One hour after the challenge, skin areas were photographed, and the thickness of the ears was measured with a dial thickness gauge after which the mice were euthanized. The dye was extracted from dissected ears in 700 μl of formamide for 12 hours at 62°C and quantitated by a spectrophotometer at 620 nm.

For the allergic asthma mouse model, 24 female BALB/c mice were randomly divided into four groups: blank group, OVA group, SiO_2_ group, and OVA+SiO_2_ group. The mice were sensitized on Days 0, 7, and 14 by an intraperitoneal injection of 50 μg of OVA (Sigma–Aldrich, Grade V) emulsified with 4 mg of aluminum hydroxide (Thermo Scientific) in 200 μL of phosphate-buffered saline (PBS). On Days 15 to 21, mice in the SiO_2_ and OVA+SiO_2_ groups were exposed to SiO_2_ solution (500 μg/mL, 50 µl per mouse) *via* intranasal instillation. On Days 19-21, mice in the OVA and OVA+SiO_2_ groups were challenged with a 1% (W/V) OVA solution *via* intranasal instillation.

### Measurement of airway hyperresponsiveness

Twenty-four hours after the last intranasal OVA challenge, airway hyperresponsiveness (AHR) in response to inhaled aerosolized methacholine was measured using the FlexiVent system (SCIREQ, Montreal, Canada) following the manufacturer’s protocol. The mice were subjected to tracheostomy and endotracheal intubation after anaesthetization with 50 mg/kg pentobarbital sodium (Sigma–Aldrich, St. Louis, USA) by intraperitoneal injection, and then the mice were connected to a ventilator. Aerosolized normal saline (0.9% NaCl) or a dose of methacholine (Sigma–Aldrich, St. Louis, USA) (6.25, 12.5, 25, and 50 mg/mL) was administered to the mice *via* a nebulizer. AHR was assessed by measuring the changes in lung resistance.

### Measurements of IgE, cytokines, histamine, and tryptase in BALF and serum

After measurement of AHR, bronchoalveolar lavage fluid (BALF) was collected by instilling and retrieving 0.5 mL of cold PBS into the lung tissues three times. Then, BALF was centrifuged at 1500 rpm for 10 min at 4°C, and supernatants were stored at −80°C until further use. The cell pellets were resuspended in 50 µL of sterile PBS to calculate the total cell counts using the TC20™ Automated Cell Counter (Bio–Rad Laboratories, Inc.). The levels of IL-4 and IL-6 in BALF supernatant were detected using a commercial ELISA kit (Multisciences (Lianke) Biotech Co., Ltd, Hangzhou, China) according to the manufacturers’ instructions. Blood was collected at the end of the experiment, and serum was acquired after allowing the blood to stand at room temperature for at least 2 hours. The levels of OVA-specific IgE (Cusabio Technology LLC, Wuhan, China), histamine (Elabscience Biotechnology Co., Ltd., Wuhan, China), and tryptase (Cusabio Technology LLC, Wuhan, China) in BALF and serum were measured using a commercial ELISA kit according to the manufacturers’ instructions.

### Lung histology and immunohistochemistry

For histological analysis, the left lung tissues were fixed in 10% neutral-buffered formalin (Biomics Biotech, Nantong, China). Paraffin-embedded lung tissues (5 mm thick) were subjected to hematoxylin and eosin (H&E) and periodic acid-Schiff (PAS) staining to evaluate tissue inflammation and goblet cell metaplasia. Histological scores were determined by randomly selecting 6-8 different fields under a microscope and evaluated by a pathologist. The numbers of peribronchial and perivascular infiltrating inflammatory cells were scored as follows: 0, no cells; 1, a few cells; 2, a ring of inflammatory cells, one cell layer of peribronchial cells; 3, a ring of inflammatory cells, two to four cell layers of peribronchial cells; and 4, a ring of inflammatory cells, more than four cell layers of peribronchial cells. The mucus hypersecretion score by PAS staining was determined as follows: the percentage of the mucus-positive area of the whole bronchus was 0, ≤5%; 1, 5%-25%; 2, 25%-50%; 3, 50%-75%; and 4, >75%.

Immunohistochemistry of mast cell tryptase in lung tissue was performed using an anti-mast cell tryptase antibody (Affinity Biosciences, cat.DF6758). Heat-mediated antigen retrieval was performed after deparaffinization. The tissue sections were blocked with goat serum. Then, the tissue sections were incubated with 1 μg/mL rabbit anti-mast cell tryptase antibody overnight at 4°C. The sections were incubated with horseradish peroxidase (HRP)-conjugated secondary antibody at 37°C for 40 min. The tissue sections were developed using Streptavidin-Biotin-Complex (SABC) with diaminobenzidine (DAB) as the chromogen.

### Transmission electron microscopic analysis

For TEM analysis, mast cells were fixed with a fixative solution containing 2% paraformaldehyde and 2% glutaraldehyde in 0.1 M phosphate buffer (pH 7.4). Cells were then postfixed in 1% OsO4 at 4°C for 2 hours. After rinsing the fixative solution from the cells, the samples were dehydrated with a graded series of ethanol and embedded in epoxy resin. Thin sections were cut with a diamond knife and mounted on copper grids. The grids were stained for 30 min each in Reynolds’s lead citrate and aqueous uranyl acetate. Ultrathin sections (thickness 70 nm) were observed under an electron microscope (HT7700, HITACHI, Japan) at 80 kV.

### Scanning transmission X-ray microscopy, STXM

The mast cells were detached from the culture medium and washed with phosphate-buffered saline (PBS) three times. Then, they were fixed with 10% neutral formalin for 10 min and dehydrated by a graded dehydration series of ethanol solution, including 70% (40 min), 80% (15 min), 90% (10 min), and 100% (10 min) ethanol solutions, at room temperature (19). Finally, the cell suspension was dropped onto a special microporous gold mesh (UltrAuFoil, Ted Pella Inc.). The mesh size was 2 μm, and the thickness of the gold mesh was only 50 nm, so soft X-rays could still be effectively transmitted. The key question of the possible intake of silicon dioxide nanoparticles by mast cells was studied by scanning transmission microscopy (STXM) at beamline station BL08U1a at Shanghai Synchrotron Radiation Facility (SSRF) (20). The microscope used a soft X-ray in the energy ranging from150-2000 eV to image a sample. The dual-energy imaging method was used in the experiment (21), whereby the value of dual-energy was set as 1840 eV for pre-edge and 1848 eV for on-the-edge. The energy value was calibrated against the reference spectrum recorded from a standard sample (nano-SiO_2_).

### Flow cytometry detection

Mature BMMCs were collected in 6-well plates. Nano-SiO_2_ was incubated with non-IgE-sensitized BMMCs for 12 hours and then added APC anti-mouse FcϵRIα antibody (Biolegend, CA, USA) to detect the effect of nano-SiO_2_ exposure on the expression of FcϵRI in unsensitized mast cells. Similarly, BMMCs were first incubated with anti-DNP IgE to sensitize, washed to remove unbound anti-DNP IgE, then treated with nano-SiO_2_ and anti-DNP IgE, and finally added APC anti-mouse FcϵRIα antibody (Biolegend, CA, USA) and FITC anti-mouse IgE antibody (Biolegend, CA, USA) to detect the effect of nano-SiO_2_ exposure on FcϵRI expression and IgE binding in IgE-sensitized mast cells.

### Transcriptome sequencing

Mature BMMCs were collected after treatment with nano-SiO_2_ particles for transcriptome sequencing analysis. The experiment was divided into four groups: blank control, DNP-HSA, SiO_2_, and DNP-HSA+SiO_2_. We performed 2×150 bp paired-end sequencing (PE150) on an Illumina Novaseq™ 6000 (LC-BioTechnology CO., Ltd., Hangzhou, China) following the vendor’s recommended protocol. Bioinformatics analysis was performed using the OmicStudio tools at https://www.omicstudio.cn/tool.

### Western blotting

Western blotting was used to investigate the effects of nano-SiO_2_ particles on the activation of the FcϵRI signaling regulatory proteins LYN and Plcγ1 and the MAPK signaling proteins P38, JNK, ERK1/2, and NF-κB. BMMCs were sensitized with 500 ng/mL DNP-IgE overnight, washed twice with Tyrode’s buffer, and placed in Tyrode’s buffer. After incubating with or without nano-SiO_2_ particlesat 37°C for 1 hour, BMMCs were stimulated with 100 ng/mL DNP-HSA for 5 to 30 min. Cells were lysed in RIPA buffer (Beyotime, Beijing, China) with protease inhibitor cocktail (Med Chem Express, Monmouth Junction, NJ) and phosphatase inhibitor cocktail (Beyotime, Beijing, China). Cell lysates were centrifuged at 12,000 rpm for 15 min. Supernatants were mixed with a loading sample buffer (Thermo Fisher Scientific) and denatured by heating for 10 min at 100°C. Proteins were separated by sodium dodecyl sulfate-polyacrylamide gel electrophoresis and transferred to polyvinylidene difluoride membranes (Merck Millipore, Billerica MA). After incubation with a primary antibody in Tris-buffered saline with 0.1% Tween 20 buffer that containing 5% bovine serum albumin, the membranes were incubated with horseradish peroxidase-conjugated secondary antibodies for 1 hour at room temperature with shaking. Chemiluminescence reagents (Biosharp, Hefei, China) were applied according to the manufacturer’s protocol. Western blot images were captured using a Tanon 5200 Multi chemiluminescent imaging system (Tanon Science & Technology Co., Ltd, Shanghai, China).

### Statistical analysis

Descriptive parameters, such as the means and standard deviations (SD) and the frequencies and percentages for categorical data, were calculated. For normally distributed data, the independent Student’s t test or one-way analysis of variance (ANOVA) was used to compare different groups. *P*<0.05 was considered statistically significant. Analyses were performed using GraphPad Prism version 8.0 software (GraphPad Software, Inc., San Diego, CA, USA).

## Results

### Cytotoxicity of particle exposure on mast cells

**Figure 1:**
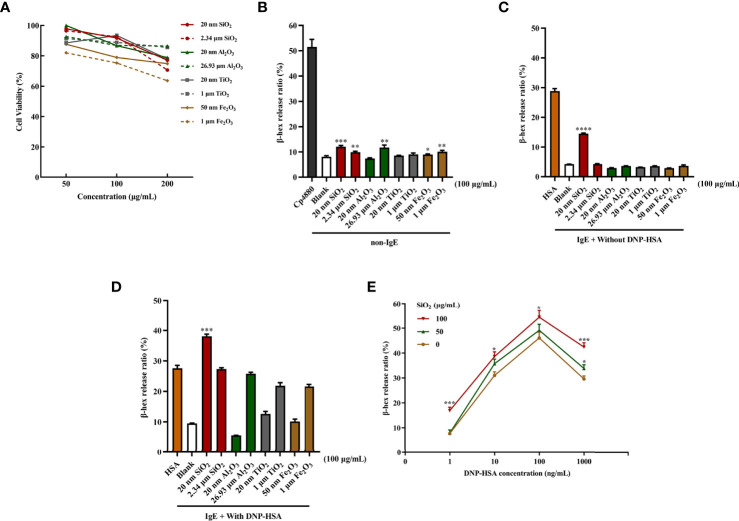
Particle exposure induced cytotoxicity and degranulation of mast cells. **(A)** Cytotoxicity of particle exposure on mast cells. **(B)** Non-IgE pathway-mediated mast cell activation. BMMCs were not sensitized with anti-DNP IgE and directly treated with particles of different sizes. Compound 48/80 was used as the positive control. The comparator was the blank control. **(C)** BMMCs were sensitized with anti-DNP IgE before treatment with different particle sizes but without DNP-HSA stimulation. DNP-HSA stimulation was used as the positive control. The comparator was the blank control. **(D)** IgE-mediated mast cell degranulation. BMMCs were sensitized with anti-DNP IgE before treatment with particles of different sizes and stimulated with DNP-HSA. The comparator was the DNP-HSA stimulation group. **(E)** Effects of different doses of 20 nm SiO_2_ exposure on FcϵRI-mediated mast cell degranulation. All data are presented as the mean ± SD. The unpaired t test or ordinary one-way ANOVA was used to compare different groups. *P < 0.05, **P < 0.01, ***P < 0.001, ****P < 0.0001.

First, we determined whether the different-sized particles posed toxic effects on mast cells. BMMCs were incubated with different doses of particles of different sizes (0 to 200 μg/mL). As shown in **Figure 1** **A**, the results suggested that exposure to high doses (200 μg/mL) of the particles could induce pronounced toxicity to mast cells. In contrast, some particles at low doses (50 and 100 μg/mL) showed minimal toxic effects on BMMCs. Among the particles, both 50 nm and 1 μm Fe_2_O_3_ showed strong cytotoxicity with increasing concentration.

### Particle exposure to non-IgE- and IgE-mediated mast cell degranulation

Mast cell degranulation was assayed by measuring the release of β-hexosaminidase by mast cells into the culture supernatants. For non-IgE pathway-mediated mast cell activation, compared with the blank control, SiO_2_ (20 nm and 2.34 μm), Al_2_O_3_ (26.93 μm), and Fe_2_O_3_ (50 nm and 1 μm) showed stimulatory effects on mast cell activation with statistical significance at 100 μg/mL. However, the activation effects were very slight to a certain degree, while Fe_2_O_3_ probably had a cytotoxic effect on mast cells (**Figure 1** **B**). The activation effects were even milder at low concentrations (25 and 50 μg/mL). Conversely, for IgE-mediated mast cell degranulation, the BMMCs were sensitized with anti-DNP IgE before the addition of the particles at different doses. Compared with the blank control, only the 20 nm SiO_2_ particles at 100 μg/mL significantly activated mast cells after IgE sensitization, improving the activation rate by approximately 10%, while other particles stimulation alone did not (**Figure 1** **C**). Interestingly, when IgE-sensitized mast cells were first incubated with the particles and then activated by the addition of DNP-HSA, compared with the DNP-HSA stimulation positive control, only the 20 nm SiO_2_ particles at 100 μg/mL significantly increased the release of β-hexosaminidase, improving the activation rate by over 10%, while other particles’ stimulation alone did not (**Figure 1** **D**). However, at low concentrations (25 and 50 μg/mL), none of the particles with or without DNP-HSA stimulation significantly activated mast cells. In addition, a dose-response relationship was observed for 20 nm SiO_2_ particles exposure with increasing DNP-HSA stimulating concentrations. Mast cells showed the most significant activation effect at a 20 nm SiO_2_ particles concentration of 100 μg/mL and DNP-HSA concentration of 100 ng/mL (**Figure 1** **E**).

### Nano-SiO_2_ particles Promote IgE-mediated PCA mouse model

**Figure 2:**
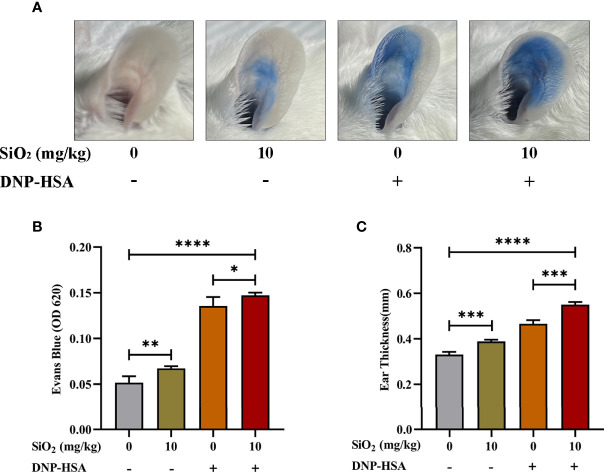
Nano-SiO_2_ particles promote IgE-mediated PCA in mice. **(A)** Representative images of PCA mouse ears. **(B)** Nano-SiO_2_ particles increase PCA-induced Evan’s blue extravasation (OD620nm) and **(C)** promote PCA-induced ear thickening. The data are presented as the mean ± SD. The unpaired t test or ordinary one-way ANOVA was used to compare different groups. *P < 0.05, **P < 0.01, ***P < 0.001, ****P < 0.0001.

We used an IgE-mediated PCA model to examine the effects of nano-SiO_2_ particles on IgE-mediated allergic reactions *in vivo*, which was quantified by the amount of Evan’s blue dye in the IgE-sensitized mouse area. As shown in **Figure 2**, compared with the blank group, Evan’s blue extravasation (**Figures 2** **A, B**) and ear thickness (**Figure 2** **C**) in mice were significantly increased after treatment with nano-SiO_2_ particles and DNP-HSA, demonstrating vascular hyperpermeability. Compared with the DNP-HSA group, the nano-SiO_2_ particles treatment significantly enhanced Evan’s blue extravasation.

### Nano-SiO_2_ particles exposure exacerbates OVA-induced allergic asthma

We used an OVA-induced allergic asthma mouse model to further verify whether nano-SiO_2_ particles could activate mast cells *in vivo* and the effects of nano-SiO_2_ particles on the development of OVA-induced allergic asthma airway inflammation in mice. Several parameters related to airway inflammation and bronchial hyperresponsiveness were determined to evaluate the impact of nano-SiO_2_ particles exposure on the morphological abnormality and dysfunction of the airway.

**Figure 3:**
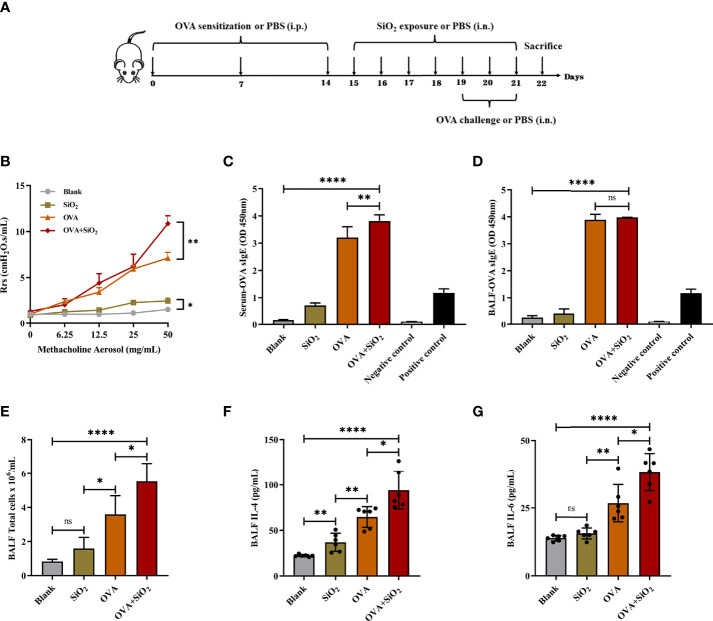
Effect of 20 nm SiO_2_ exposure on the allergic asthma mouse model. **(A)** The schedule for the OVA-induced asthma model and the procedure of intranasal instillation of 20 nm SiO_2_ or PBS in the OVA-induced asthma model. **(B)** Measurement of airway hyperresponsiveness in the OVA-induced asthma model. The values of respiratory system resistance (Rrs) were measured 24 h after the last OVA challenge by exposure to increasing doses of methacholine. Data are presented as the mean ± SEM. **(C, D)** Levels of OVA-specific IgE in BALF and serum. **(E)** Total cell counts in BALF of the OVA-induced asthma model. Data are presented as the mean ± SD. **(F, G)** Levels of IL-4 and IL-6 in BALF supernatant of the OVA-induced asthma model. Data are presented as the mean ± SD. The unpaired t test or ordinary one-way ANOVA was used to compare different groups. *P < 0.05, **P < 0.01, ****P < 0.0001. ns, no significance.

Airway hyperresponsiveness (AHR) was measured after the last intranasal OVA challenge. Compared with the PBS-treated blank group, airway resistance (Rrs) values were significantly elevated to various degrees in the SiO_2_ exposure, OVA, and OVA+SiO_2_ groups (**Figure 3** **B**). In particular, the Rrs values were significantly increased in the OVA+SiO_2_ compared with the OVA group (*P*<0.01). These data suggested that exposure to nano-SiO_2_ particles could affect airway function and increase airway resistance.

**Figure 4:**
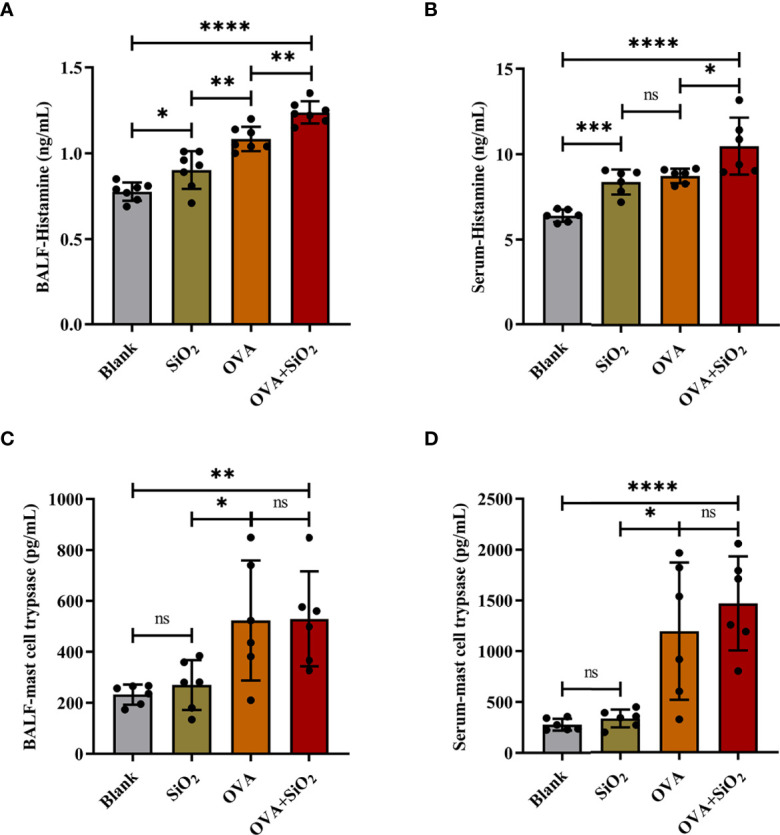
The levels of histamine and mast cell tryptase in BALF and serum of the OVA-induced asthma model. Data are presented as the mean ± SD. The unpaired t test or ordinary one-way ANOVA was used to compare different groups. *P < 0.05, **P < 0.01, ***P < 0.001, ****P < 0.0001. ns, no significance.

IgE, cytokines, and histamine are important inflammatory transmitters and activators in allergic respiratory diseases. The levels of OVA-specific IgE (sIgE), histamine, and tryptase in BALF and serum were measured. As shown in **Figure 3**, compared with the OVA alone group, the levels of OVA-sIgE in serum were significantly increased when exposed to OVA+SiO_2_, although a similar trend was not observed in BALF (**Figures 3** **C, D**). Moreover, the total BALF inflammatory cells in allergic asthma mice exposed to nano-SiO_2_ particles were significantly higher than those in the blank and OVA groups (**Figure 3** **E**). The levels of IL-4 and IL-6 in the BALF supernatant were detected. Nano-SiO_2_ particles exposure significantly increased IL-4 and IL-6 in BALF between the OVA group and OVA+SiO_2_ group (**Figures 3** **F, G**). In addition, compared with that in the blank control group, IL-4 secretion, but not IL-6 secretion, was elevated in the SiO_2_ exposure group. Histamine levels in both serum and BALF were increased to varying degrees after exposure to nano-SiO_2_ particles compared with the blank control. Moreover, histamine was also significantly higher in the OVA+SiO_2_ group than in the OVA alone group (**Figures 4** **A, B**). However, exposure to nano-SiO_2_ particles did not significantly increase the level of tryptase secretion, nor did it show a difference between the OVA alone group and the OVA+SiO_2_ group (**Figures 4** **C, D**).

**Figure 5:**
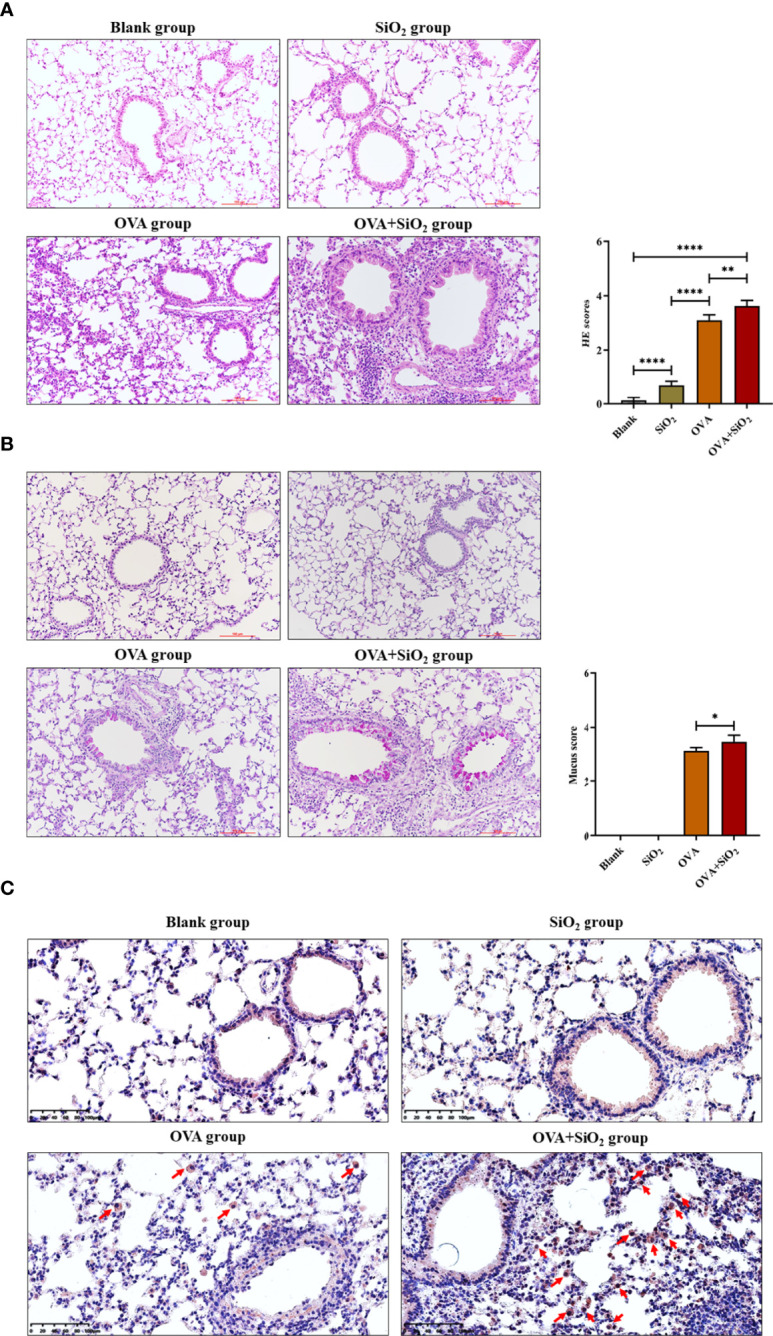
Representative images of lung histology and immunohistochemistry. **(A)** Lung tissue stained with hematoxylin and eosin (H&E) (× 200). **(B)** Lung tissue stained with periodic acid-Schiff stain (PAS) (× 200). **(C)** Immunohistochemistry analysis of mast cell tryptase in lung tissue. The arrow indicates anti-mast cell tryptase antibody-positive cells. Data are represented as the means ± SD. The unpaired t test was used to compare different groups. *P < 0.05, **P < 0.01, ****P < 0.0001.

Representative images of lung histology and immunohistochemistry are shown in **Figure 5**. The blank control group exhibited a normal lung tissue structure and clear pulmonary alveoli. The SiO_2_ exposure group showed slight congestion of the alveolar walls, slight thickening of the bronchiolar walls, and a small amount of inflammatory cell infiltration around the bronchioles. Histological analysis showed peribronchial and perivascular inflammation in all OVA-treated groups. OVA+SiO_2_ exposure induced more serious alveoli and bronchial injuries than OVA exposure alone. The inflammatory injury was characterized by hemorrhage, edema, a thickened alveolar wall, and pronounced inflammatory infiltrates within the interstitial and alveolar spaces. The alveolar cavity and bronchioles were structurally collapsed, and epithelial cells were destroyed, with more goblet cell hyperplasia. After exposure to nano-SiO_2_, airway inflammation and mucus hypersecretion scores were significantly elevated in the OVA+SiO_2_ group (**Figures 5** **A, B**). Moreover, the immunohistochemistry analysis of mast cell tryptase in lung tissue showed that the anti-mast cell tryptase antibody-positive cells had more aggregation in the OVA+SiO_2_ group than in the OVA alone group (**Figure 5** **C**). Taken together, our data indicated that nano-SiO_2_ particles exposure exacerbated allergic airway inflammation, triggered goblet cell proliferation, recruited mast cells, increased histamine secretion, and enhanced airway responsiveness.

### STXM and TEM analyses show that nano-SiO_2_ can enter the mast cells

**Figure 6:**
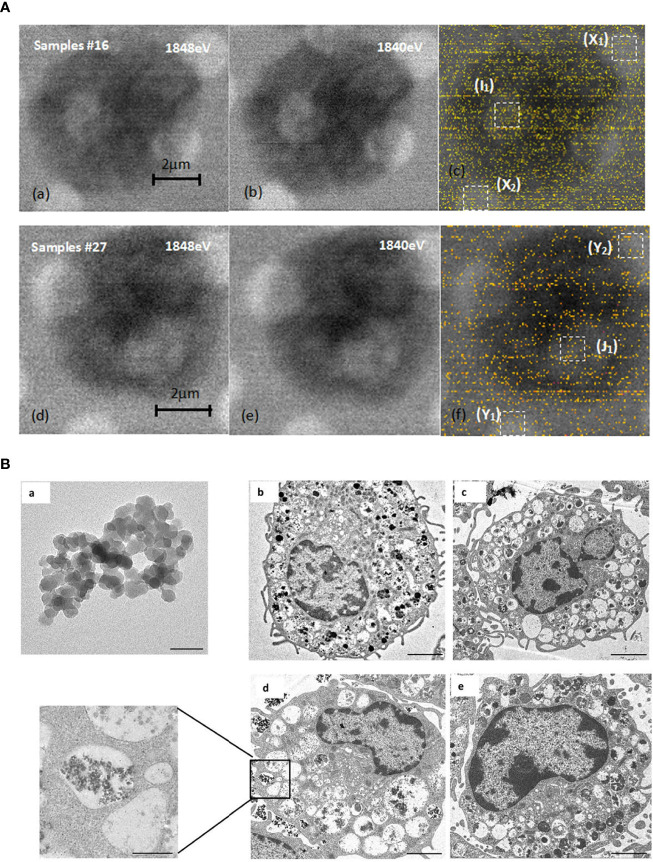
STXM and TEM analysis. **(A)** Comparison of dual-energy imaging between cells (samples #16) loaded with SiO_2_ nanoparticles, images shown in **(a)**, **(b)**, **(c)**, and empty cells (sample #27), with the images shown in **(d, e, f)**. **(B)** SiO_2_ promoted mast cell degranulation. **(a)**. Transmission electron microscope image of 20 nm SiO_2_ (×1000 k, bar=50 nm). **(b–e)**. Representative transmission electron microscope images of mast cells. b: Blank group (×2.5 k, bar=2 μm); c: DNP-HSA group (×2.0 k, bar=2 μm); d: DNP-HSA+SiO_2_ group (×2.5 k, bar=2 μm; ×10 k, bar=500 nm); e: SiO_2_ group (×3.0 k, bar=2 μm).

The results of dual-energy imaging of the two samples are shown in **Figure 6** **A**. Sample 16 was a dehydrated cell with silica particles (**Figure 6A** **a, b, c**), and sample 27 was an empty cell for comparison (**Figure 6A** **d, e, f**). Fig. (c) and (f) show the derived results of dual-energy imaging with diffuse distributions of yellow dots. Because the energy of the silicon K-edge was close to the upper limit of the energy range of the beamline, the light flux dropped to a low level. The counting rate of the signals was low, which led to a poor signal-to-noise ratio in the processed images, so it was impossible to obtain a clear and distinguishable element distribution map. Nevertheless, a statistical analysis of the frequency of occurrence of likelihood (FOL), “yellow dots” in the dual-energy map, could provide helpful hints as to whether SiO_2_ particles were included in the sample. A statistical box with a size of 1 μm × 1 μm, shown as white-dashed boxes in the figure, was used in the analysis. They were chosen to be positioned over mesh holes where X-rays have better penetration and therefore more effective signals for silicon content. From the direct-scanned images of Figure (d) and (e), it could be seen that there was no cell coverage on the golden mesh in the Y1 and Y2 areas, so the FOL should be mainly from the background noise. The gold mesh in area J1 was covered with cells, but its FOL was the same as that for the bare area within the error bar of the statistical range (**Table 1**). This result suggested that sample 27 was an empty cell. The same method was used to analyze sample 16 (Figure c). The direct-scanned images are in **Figure 6** **Aa, b**) revealed no obvious cell sample coverage on the golden mesh in areas X1 and X2, and the FOLs for both were relatively close (**Table 1**), which could be considered background noise. The gold mesh of the I1 region was covered with cells, and a high FOL was recorded, which was almost doubled, strongly suggesting that the cells were loaded with SiO_2_ particles. Furthermore, we noted that the background noise for sample16 was greater than that for sample 27, which was related to the instrument and did not necessitate cross-checking. To summarize the dual-energy imaging results, clear elemental distribution mapping was not possible due to the low signal-to-noise ratio. Nevertheless, a statistical analysis of loaded cell samples demonstrated a high FOL number in the area covered by cells, suggesting the presence of silicon nanoparticles in the cells.

**Table 1:** Statistical analysis of FOL on samples at various areas marked by white-dashed boxes.

Sample	27 (empty cell)			16 (cell with silica particles)		
Area	Y1	Y2	J1	X1	X2	I1
FOL	13 ± 2	13 ± 2	17 ± 2	30 ± 2	36 ± 2	71 ± 2

FOL, frequency of occurrence of likelihood.

We further investigated whether nano-SiO_2_ particles could enter mast cells and promote degranulation. The morphology of mast cells and SiO_2_ was investigated by transmission electron microscopy (TEM). Compared with the control group, the cells treated with DNP-HSA or DNP-HSA+SiO_2_ exhibited obvious morphological changes, such as deformation, shrinkage, secretory granule release, sparse intracellular particles, and vacuolated cytoplasm, altogether indicating the occurrence of degranulation (**Figure 6** **B**) (22). However, there was no clear morphological change in mast cells in the SiO_2_ group, which seemed to indicate that treatment with SiO_2_ alone was insufficient to cause obvious degranulation. Compared with the DNP-HSA group, an increased degree of intracellular vacuoles was observed in DNP-HSA+SiO_2_. Moreover, we found nano-SiO_2_ particles inside the mast cells. These results showed that nano-SiO_2_ particles could enter the mast cells and exert a synergistic sensitization effect through the IgE pathway.

### Effects of nano-SiO_2_ exposure on the gene expression profile in mast cells

To deeply investigate the details of the nano-SiO_2_ particles effects, we examined the gene expression profile alterations after nano-SiO_2_ particles treatment in BMMCs. The difference analysis between the groups showed that 285 genes were significantly expressed after treatment with nano-SiO_2_ particles. The differentially expressed genes were further searched and analyzed in the GeneCards database, and 135 differentially expressed genes were found to be related to oxidative stress, suggesting that the exposure of nano-SiO2 particles may cause oxidative stress in mast cells.

**Figure 7:**
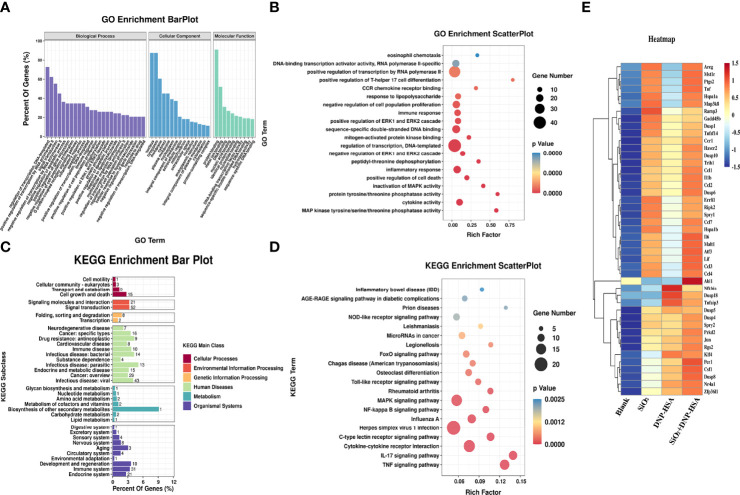
Nano-SiO_2_ exposure affects the gene expression profile. **(A)** Gene Ontology (GO) enrichment barplot analysis. **(B)** GO enrichment scatterplot analysis. **(C)** Kyoto Encyclopaedia of Genes and Genomes (KEGG) pathway enrichment barplot analysis. **(D)** KEGG pathway enrichment scatterplot analysis. **(E)** Heatmap of clustering analysis for differentially expressed genes related to signaling pathway.

Gene Ontology (GO) and Kyoto Encyclopedia of Genes and Genomes (KEGG) pathway enrichment analyses were used to analyze these differentially expressed genes. As shown in **Figure 7** **A**, in terms of biological process, differentially expressed genes were mainly enriched in transcriptional regulation, signal transduction, apoptosis process, inflammatory response, cell proliferation, phosphorylation, and positive regulation of ERK1/2 cascade, etc. In terms of cellular component, the cellular structural positions where differentially expressed genes perform their functions were mainly located in the nucleus, cytoplasm, and cell membrane. In terms of molecular function, the activities of gene products at the molecular level mainly involved protein binding, metal ion binding, DNA and nucleic acid binding, and hydrolase activity. Further, taking the Top 20 terms with the smallest *p*-value for GO enrichment scatterplot display, GO enrichment analysis showed that the genes were enriched in MAP kinase tyrosine/serine/threonine phosphatase activity, protein tyrosine/threonine phosphatase activity, and regulation of the ERK1 and ERK2 cascades (**Figure 7** **B**). As shown in **Figure 7** **C**, the KEGG enrichment bar plot showed that the differentially expressed genes covered the KEGG Main Class, involving several secondary classifications such as cell growth and death, signal transduction, environmental adaptation, metabolism, and immune system, etc. The KEGG pathway enrichment analysis demonstrated that the differentially expressed genes were primarily involved in cytokine-cytokine receptor interactions, the NF-κB signaling pathway, and the MAPK signaling pathway (**Figure 7** **D**). We further screened differentially expressed genes related to the MAPK signaling pathway, NF-κB signaling pathway, and ERK1/2 cascade positive and negative regulation for cluster analysis. A total of 45 differentially expressed genes were screened (**Figure 7E**). Compared with the blank group, both nano-SiO_2_ exposure and DNP-HSA stimulation increased the expression of genes related to the MAPK signaling pathway in mast cells to varying degrees. Exposure to nano-SiO_2_ significantly up-regulated the expression of genes such as *Areg*, *Ptgs2*, *Tnf*, *Hspa1a*, *Map3k8*, *Malt1*, *Ccl3*, *Ccl4*, and *Il6*.

### Nano-SiO_2_ exposure enhances the MAPK signaling pathway

**Figure 8:**
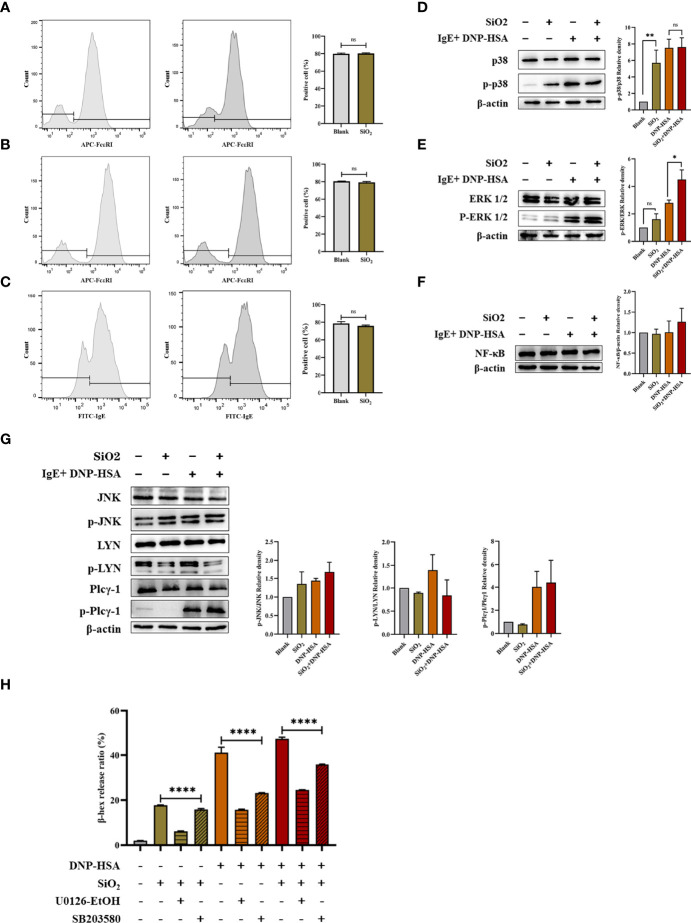
FcϵRI-mediated signaling in mast cells. **(A)** BMMCs were not sensitized with anti-DNP IgE and treated with nano-SiO_2_. The results showed no significant difference in FcϵRI expression compared to the blank control. **(B, C)** BMMCs were sensitized with anti-DNP IgE and washed to remove unbound anti-DNP IgE before treatment with nano-SiO_2_. There were no significant differences in FcϵRI expression **(B)** or IgE binding capacity **(C)** compared to the blank control. **(D–G)** BMMCs were sensitized with anti-DNP-IgE, treated with nano-SiO_2,_ and stimulated with DNP-HSA. The cell lysates were analyzed by Western blotting with different antibodies against the NF-κB proteins and the phosphorylated and nonphosphorylated forms of the p38, ERK1/2, JNK, LYN, and Plcγ1 proteins. Relative density bars represent the means ± SD. **(H)** β-hexosaminidase release ratio from activated BMMCs pretreated with signaling inhibitors (ERK inhibitor U0126-EtOH 5 μM/L and p38 inhibitor SB203580 10 μM/L). The unpaired t test or ordinary one-way ANOVA was used to compare different groups. *P < 0.05, **P < 0.01, ****P < 0.0001. ns, no significance.

First, we investigated whether nano-SiO_2_ particles affected the expression of high-affinity IgE receptors (FcϵRI) in mast cells and the ability to bind IgE. BMMCs showed no significant difference in FcϵRI expression after incubation with SiO_2_ particles compared to the blank control (**Figure 8** **A**). Then, BMMCs were sensitized by incubation with anti-DNP-IgE, washed to remove unbound IgE, and treated with SiO_2_ particles. Flow cytometry detection revealed no significant differences in FcϵRI expression or IgE binding capacity compared to the blank control (**Figures 8** **B, C**). Overall, the above data confirmed that nano-SiO_2_ particles exposure did not affect the expression of FcϵRI or the ability of mast cells to bind IgE.

Given the transcriptome sequencing analysis results, we further investigated the signaling pathways involved in mast cell activation. BMMCs were sensitized with anti-DNP-IgE, treated with nano-SiO_2_ particles, and stimulated with DNP-HSA. Cell lysates were analyzed by Western blotting with different antibodies. As shown in **Figures 8** **D–G**, the results indicated that nano-SiO_2_ particles treatment upregulated p-ERK1/2 expression based on DNP-HSA stimulation. Nano-SiO_2_ particles treatment increased p-p38 expression compared with the blank control, while this trend was not observed after DNP-HSA stimulation. The expression of NF-κB, p-Plcγ1 and p-JNK was upregulated after mast cell activation, but no significant differences were observed between the DNP-HSA stimulation group and the DNP-HSA+SiO_2_ group. Taken together, nano-SiO_2_ particles stimulation may synergistically activate mast cells by enhancing the MAPK signaling pathway, especially the phosphorylation levels of ERK1/2.

Next, we validated using the MAPK signaling inhibitors (ERK inhibitor U0126-EtOH and p38 inhibitor SB203580). As shown in **Figure 8** **H**), when the BMMCs were pretreated with signaling molecule inhibitors, the β-hex release ratio of the SiO_2_ group, the DNP-HSA group, and the DNP-HSA+SiO_2_ group all decreased to varying degrees, and the differences were statistically significant (*P*<0.0001). Compared with the 10 μM/L p38 inhibitor, the inhibitory effects of the 5 μM/L ERK inhibitor were more obvious, and the β-hex release ratio was reduced by about 10%-25%.

## Discussion

An increasing number of observational studies have demonstrated that air pollution has an adverse impact on allergic respiratory disease. Air pollution exposure is associated with increased asthma and allergy morbidity and is a suspected strong risk factor for the increasing prevalence of allergic conditions (23–25). However, how the different components or PM with different particle sizes in air pollution affect the allergic response is unclear. In the present study, we explored the effects of four common mineral elements with different particle sizes in smog particles on mast cell activation. The results showed that nano-SiO_2_ particles stimulation might synergistically activate IgE-sensitized mast cells by enhancing the MAPK signaling pathway, and nano-SiO_2_ particles exposure could exacerbate allergic inflammation in an OVA-induced asthmatic mouse model.

Mast cells play an essential role in various allergic disorders, acting as the first line of defense in response to external environmental stimuli (13). Activated mast cells release various mediators, including histamine and inflammatory cytokines in response to various stimuli (26). Numerous studies have confirmed that PM2.5 can promote mast cell degranulation, increase the release of inflammatory cytokines, and aggravate allergic diseases (27–29). However, only a few studies have examined the effects of pollutant components on allergic diseases because pollutants derive from different sources and vary in composition and toxicity (30). In our previous study, nine typical water-soluble inorganic salts (ammonium nitrate, ammonium sulfate, ammonium chloride, sodium nitrate, anhydrous sodium sulfate, potassium nitrate, potassium sulfate, calcium nitrate, and calcium sulfate) and three water-soluble organic acids (malonic acid, succinic acid, oxalic acid) were selected in PM2.5 to investigate the cytotoxicity and activation effect on mast cells. These results indicated that nine typical water-soluble inorganic salts could not induce the release of β-hex on BMMCs through either the IgE pathway or the non-IgE pathway (16). In water-soluble organic acids, only malonic acid was observed to have a very weak activation effect on BMMCs. In the present study, we found that different particle sizes of Al_2_O_3_, Fe_2_O_3_, and SiO_2_ showed a slight activation effect on mast cell β-hex release in the non-IgE pathway. We also found that SiO_2_ at 20 nm was able to increase β-hex release from IgE-sensitized mast cells, while Al_2_O_3_, TiO_2_, Fe_2_O_3_, and SiO_2_ (2.34 μm) did not. Depending on their size and mass, particles can reach different sites within the respiratory tract. For example, PM10 can enter the upper airways, while finer particles, such as PM2.5 and PM0.1, can enter the terminal bronchioles and alveoli. If the particles are small enough, they can enter the blood, which in turn can affect other organ systems (31, 32). In our experiments, due to the different masses of particulate matter, its sedimentation speed in the medium was different, resulting in a difference in the time of sufficient contact with mast cells. In addition, there may also be differences in the uptake capacity of mast cells to different particle sizes. However, the TEM and STXM results showed that 20 nm SiO_2_ particles could enter the mast cells and exert a sensitizing effect. Importantly, we cannot ignore the effect of the protein composition of the cell culture medium on particulate matter. Particles may be bound or coated by bioactive proteins in the medium, which not only impacts the behavior of nanoparticles but also affects the bound proteins. Therefore, in our experiments, only nano-SiO_2_ particles was observed to promote the activation of IgE-sensitized mast cells, but this did not exclude those other particles that would not affect mast cells. In fact, several studies have investigated the effects of the chemical composition and redox activity of particles on exacerbating allergic airway sensitization and the type of immune response (33–36). Although our study attempted to investigate the effects on mast cells from single particles of different sizes, further research is needed to understand the immune-modulatory effects of different particles in pollutants and the impact of particle material, other sizes, and surface coating.

Given the results of the *in vitro* cellular experiments, we further verified the effect of nano-SiO_2_ particles on allergic diseases *in vivo*. The PCA ear model is a typical acute allergic animal model wherein allergic reactions are induced by antigen stimulation of mast cells in ear skin, which is widely used in the screening of anti-allergic drugs and drug safety assessment (37, 38). In our PCA mouse model, Evan’s blue extravasation in mice was significantly increased after treatment with nano-SiO_2_ particles, which indicated that the nano-SiO_2_ particles exposure might promote the local IgE-sensitized mast cell activation and allergic mediators’ secretion, thereby increasing the permeability of local blood vessels and aggravating allergic reactions. Coexposure to nano-SiO_2_ particles plus OVA sensitization caused an enhancement of allergic airway inflammation compared with OVA alone. This adjuvant-like effect was manifested by significantly greater OVA-specific serum IgE, airway hyperresponsiveness, lung inflammation injury, mucous cell metaplasia, and cytokine expression compared with mice sensitized to OVA without nano-SiO_2_. Our findings were consistent with those of other studies. Han et al. reported that silica nanoparticles with OVA induced more significant inflammatory cell infiltration in BALF, extensive pathological changes, and higher cytokine levels than silica nanoparticles alone or saline/OVA. In particular, mesoporous-type silica nanoparticles showed the most severe airway inflammation in both direct toxicity and adjuvant effect assays (39). Brandenberger et al. suggested that engineered silica nanoparticles promoted the immunologic response towards the allergen and thereby potentiated the adverse allergic responses in the pulmonary airways, and the adjuvant effects of silica nanoparticles were Th2/Th17 related (40). One murine model experiment showed that SiO_2_ induced epithelial cells to express IL-33, which in turn activated innate lymphoid cells to produce IL-5 and/or IL-13, contributing to the exacerbation of OVA-induced airway inflammation (41). Another study indicated that silica nanoparticles aggravate airway inflammation and asthma development by increasing the protein expression levels of thioredoxin-interacting protein (TXNIP) and the NOD-like receptor pyrin domain-containing 3 (NLRP3) inflammasome (42). However, Shin JH et al. conducted a subacute inhalation toxicity study of synthetic amorphous silica nanoparticles at low, middle, and high concentrations in rats using a nose-only inhalation system. They did not observe any toxic effects on the lungs of rats at any concentration (43). In Horie M’s study, SiO_2_ nanoparticles did not affect OVA-specific IgE and IgG1 levels and did not show the potential to aggravate allergic reactions (36).

Obviously, the effect of SiO_2_ particles exposure on the body is a very complex process, and the airway epithelial cells and alveolar macrophages are undoubtedly the most concerned. Studies have pointed out that occupational mineral dusts and air pollutant particles can induce airway wall remodeling (44, 45). Using the human airway epithelial cells 16HBE or primary cultured mouse tracheobronchial epithelial cells, researchers found that SiO_2_ nanoparticles could inhibit the responses to ATP and inhibit cation channel transient receptor potential vanilloid 4 (TRPV4) in airway epithelial cells, and result in epithelial barrier dysfunction (46, 47). *In vitro* and *in vivo* experiments demonstrated that SiO_2_ particles exposure not only caused rapid NLRP3-dependent mitochondrial depolarization and DNA damage in airway epithelial cells but also led to ultrastructural defects in airway cilia and mucus hypersecretion (48, 49). Besides, one review concluded that SiO_2_ particles might bind to the scavenger receptor of alveolar macrophages, followed by particle endocytosis with a respiratory burst to generate reactive oxygen species and reactive nitrogen species, which in turn activation of the protein kinase C mediated MAPK signaling cascades resulting in cytokine release (50). In our experiments, we attempted to evaluate the effect of nano-SiO_2_ on allergic asthma from the perspective of mast cells *in vivo* based on *in vitro* cell experiments that confirmed the activation of nano-SiO_2_ on mast cells. High inhalation doses of silica result in pulmonary fibrosis and silicosis, and silica exposure can activate various immune cells and cause a proinflammatory response (51, 52). We selected a relatively low-concentration nano-SiO_2_ exposure *in vivo* to avoid masking its synergistic sensitizing effect due to the apparent toxic reaction. Compared with the blank control group, the nano-SiO_2_ exposure alone group exhibited relatively mild pathological changes in the lung tissue, yet some changes persisted in lung function, histamine, and inflammatory factors. Compared with the OVA group, the results showed that nano-SiO_2_ exposure resulted in the accumulation of tryptase-positive cells in the airways and increased BALF histamine levels, which is associated with immediate allergic symptoms. These results suggested that airway exposure to nano-SiO_2_ could exacerbate allergic airway inflammation, trigger mast cells, increase histamine secretion, enhance airway responsiveness, and foster more serious manifestations of allergic airway disease.

Finally, we studied the effect of nano-SiO_2_ exposure on FcϵRI-mediated signaling. *In vitro*, we found that nano-SiO_2_ had a very slight activation effect on mast cells without IgE sensitization. However, when IgE-sensitized mast cells were first incubated with nano-SiO_2_ and then excited by the addition of DNP-HSA, the nano-SiO_2_ showed a synergistic effect, significantly increasing the release of β-hexosaminidase. Therefore, we first investigated whether nano-SiO_2_ affected the expression of FcϵRI in mast cells and the ability to bind IgE. The experimental results showed that nano-SiO_2_ exposure did not affect the expression of FcϵRI or the ability of mast cells to bind IgE but synergistically activated mast cells by enhancing the MAPK signaling pathway, especially the phosphorylation levels of ERK1/2, which was consistent with the results of transcriptome sequencing. The gene expression profile in mast cells showed the genes related to the MAPK signaling pathway in mast cells had significant changes, such as the *Gadd45b* and *Nr4a1*. The orphan nuclear receptor *Nr4a1* promoted FcϵRI-stimulated mast cell activation and anaphylaxis by counteracting the inhibitory LKB1/AMPK axis (53). *Gadd45b* belongs to the Gadd45 (growth arrest and DNA damage-inducible 45) family of proteins and is involved in environmental stress. It was reported that PM2.5 can promote IgE-mediated mast cell activation through ROS/Gadd45b/JNK axis (54).

Of note, the mechanism underlying the impact of pollutants on diseases is extremely complex. Cao et al. found that PM2.5 could increase the viability of human airway smooth muscle cells, accompanied by increased airway hyperresponsiveness through the kallikrein-bradykinin pathway (29). In addition, Liu et al. confirmed that PM2.5 triggered airway inflammation and bronchial hyperresponsiveness in mice by significantly downregulating the expression and activity of SIRT2, accelerating p65 phosphorylation and acetylation, and activating the NF-κB signaling pathway (55). Specific to mast cells, our results were consistent with Jin’s study, which showed that PM2.5 exposure enhanced FcϵRI-mediated signaling and mast cell function due to increased phosphorylation of Syk, LAT, SLP-76, PLC-γ1, Akt, ERK1/2 or p38 and activated the PI3K and MAPK pathways (56). However, Wang et al. pointed out that PM2.5 treatment activated MEKK4 and JNK1/2 but not ERK1/2 and p38, facilitating IgE-mediated mast cell activation (54). Moreover, another study on silicosis found that silica can direct mast cell activation, resulting in inflammatory mediators, and enhance IgE-mediated cytokine, chemokine, and protease production. However, these effects were thought to occur in part through mast cell scavenger receptors instead of the alteration of FcϵRI-mediated signaling events (57). The cellular mechanisms regulating hyperallergic responses caused by pollutants have not been entirely elucidated. The role of biologically and nonbiologically active components in pollutants, in addition to PM2.5 and nano-SiO_2_, in allergic diseases and the underlying molecular mechanisms, still merit attention and further research.

Silica is the most common component found naturally in the Earth’s crust and is an important component of respirable particulate matter in urban areas (58). Due to their specific physicochemical properties, silica nanoparticles are widely used in many engineering and medical fields, including fabrication, drug delivery systems, cosmetics, and food packing (59). These factors undoubtedly increase the risk of SiO_2_ exposure. The high incidence of allergic diseases and the increasing exposure factors similar to nano-SiO_2_ have brought new challenges to preventing and treating allergic diseases. Moreover, our results provide useful information for explaining the acute exacerbation of symptoms and the increase in medical treatment in patients with allergic diseases in smoggy weather. The synergistic effects of nano-SiO_2_ exposure promoting mast cell activation in smoggy weather, even in thunderstorms may be one of the reasons for the aggravation of symptoms in allergic patients. Our results also suggest the need to avoid nano-SiO_2_ exposure in daily life, especially in individuals with allergic respiratory diseases.

Although *in vitro* and *in vivo* experiments are important for understanding the potential toxicology and immune response to air pollution, our experiments still have some limitations. First, pollutant exposure dosing shows significant effects, but whether these effects represent the density of particles deposited in the airway is questionable, as also observed in similar studies (60). The effects of exposure dose, effective concentration, sedimentation rate, and diffusion rate of particles on cell experiments need to be further evaluated. Second, exposure to particulate matter may involve numerous immune cells such as epithelial cells, macrophages, dendritic cells, and lymphocytes, despite the mast cells localized in the skin, mucous membranes, and airways that are in contact with the environment. The effects of SiO_2_ on the immune system and allergic diseases may be far more complex than we have observed. Therefore, there is a need to further study the effects of nano-SiO_2_ on immune cells and the impact of nano-SiO_2_ on airborne allergens.

In conclusion, our results indicated that nano-SiO_2_ stimulation might synergistically activate IgE-sensitized mast cells by enhancing the MAPK signaling pathway and that nano-SiO_2_ exposure could exacerbate allergic inflammation. Our experimental results provide useful information for preventing and treating allergic diseases.

## Data Availability Statement

The datasets presented in this study can be found in online repositories. The names of the repository/repositories and accession number(s) can be found below: GEO under accession number GSE206630.

## Ethics Statement

The animal study was reviewed and approved by Nanjing Medical University’s Institutional Animal Care and Use Committee.

## Author Contributions

Y-SY: Investigation, Formal analysis, Data Curation, Writing - Original Draft; M-DC: Methodology, Investigation, Validation, Data Curation; AW: Resources, Investigation, Data Curation; Q-ML: Resources, Investigation; D-XZ: Resources, Investigation; YZ: Resources, Investigation; L-LM: Resources, Investigation; ML: Resources, Investigation; YS: Resources, Investigation; D-DX: Conceptualization, Supervision, Writing - Review & Editing; J-FW: Conceptualization, Supervision, Writing - Review & Editing; J-LS: Conceptualization, Supervision, Writing - Review & Editing. All authors contributed to the article and approved the submitted version.

## Funding

This work was supported by the National Natural Science Foundation of China (U1832212), Beijing Municipal Natural Science Foundation (7191008) and National Natural Science Foundation of China (12105313).

## Conflict of Interest

The authors declare that the research was conducted in the absence of any commercial or financial relationships that could be construed as a potential conflict of interest.

## Publisher’s Note

All claims expressed in this article are solely those of the authors and do not necessarily represent those of their affiliated organizations, or those of the publisher, the editors and the reviewers. Any product that may be evaluated in this article, or claim that may be made by its manufacturer, is not guaranteed or endorsed by the publisher.
